# Special Issue “Metabolic Engineering and Synthetic Biology Volume 2”

**DOI:** 10.3390/metabo11010035

**Published:** 2021-01-06

**Authors:** An N. T. Phan, Lars M. Blank

**Affiliations:** Institute of Applied Microbiology—iAMB, Aachen Biology and Biotechnology—ABBt, RWTH Aachen University, Worringerweg 1, 52074 Aachen, Germany

In times of ever-increasing demand for chemicals and the subsequent increase in CO_2_ in the atmosphere, we have to intensify our efforts to establish a circular (bio) economy. To reduce fossil resource use, renewable carbon sources including biomass and CO_2_ itself need to be used. The (bio)catalysts of choice that are able to convert these carbon sources into valuable chemicals often have to be tailored to meet the industrial requirements in titer, rate, and yield, and, hence, ultimately, in cost. While exciting examples exist, from vitamins to plastic monomers and bioplastics, the metabolic engineering of such biocatalysts is still time and cost consuming. With the improvement of biological tools and ideas for standardization, creating, and/or building new whole-cell biocatalysts becomes an ever more rapid task.

Here, we focused on metabolic engineering and synthetic biology that are driven by flux and/or metabolome approaches. Fluxes of all intracellular biochemical reaction steps are the ultimate outcome of genetic and environmental alterations. We are convinced that quantitative approaches in metabolite analysis will help to reduce the time required to establish an efficient whole-cell biocatalyst. In this Special Issue, Laviña, et al. demonstrated how metabolomics was utilized to characterize adaptive laboratory evolution *Escherichia coli* strains [1]. With the targeted strategy, metabolites in the central carbon metabolic pathways were quantified to understand the overall effect of the mutations acquired through evolution to improve 1-butanol production. From another example, Alden et al. employed an untargeted-metabolomics approach on Chinese hamster ovary (CHO) cells, the workhorse for the production of biopharmaceuticals [2]. By comparing metabolic profiles across cell lines as well as between different growth phases, the authors could identify 5-hydroxyindoleacetaldehyde, a tryptophan-derived metabolite, as an indicator of significant growth inhibition. These examples indicate that metabolomics can be used as an independent strategy to identify by products, highlight enzymatic bottlenecks, and/or find biomarkers.

Additionally, the intracellular fluxes show advantages in decipher redox cofactor imbalances, futile cycles, and the use of alternative pathways. Thermodynamically feasible reaction conditions can not only explain the phenotype observed but may also lead to genetic targets for further strain improvement and to new biochemical network designs. Therefore, the common approaches to study intracellular fluxes were also exemplified in this Special Issue. Specifically, metabolic profiling in combination with ^13^C flux redistribution has been performed on the first reported CRISPR-based genome-edited *Synechococcus elongatusn* to improve the co-production of succinate and ethylene [3]. In an interesting contribution on C1 consumption in *E. coli*, flux balance analysis has been shown to be useful in pathway interpretation regarding formaldehyde condensation with tetrahydrofolate [4]. Finally, pool influx kinetics (PIK) was employed to identify promising metabolic engineering targets by the pairwise comparison of up- and downstream ^13^C labeling dynamics concerning l-histidine production with engineered *Corynebacterium glutamicum* [5]. Together, the assessment of intracellular metabolite levels and pathway fluxes has proven valuable to decipher cellular metabolism, which further supports upgrading and streamlining the biocatalytic activity through metabolic engineering.

An essential group of metabolites that always draws a lot of attention is thioesters. At least one-third of all cellular carbon is typically metabolized through a thioester of coenzyme A (CoA) and they are involved in 5% of all enzymatic reactions [6]. From the industrial perspective, CoA thioesters are important precursors for the biosynthesis of lipids, polyketides, isoprenoids, amino acids, and numerous other bioproducts. Understanding the relevance of CoA thioesters, Ku, et al. have summarized the metabolic engineering strategies to increase acetyl-CoA flux [7]. The authors not only discussed the strategies that have been implemented to improve the native acetyl-CoA flux, but also presented the recent works on synthetic acetyl-CoA biosynthesis routes that achieve a higher stoichiometric yield of acetyl-CoA. In addition to this comprehensive review, Gonzalez-Garcia et al. showcased how to engineer the Wood–Werkman cycle, to provide propionyl-CoA and *S*-methylmalonyl-CoA, for the heterologous production of polyketides in *E. coli* [8].

Nevertheless, enzyme assembly, an interesting but also challenging strategy to control metabolic flux, was also highlighted in this Special Issue. In this approach, enzymes, substrates, or metabolites were ligand-bound or physically sequestrated into isolated compartments that bring them closer, and consequently, enhance the flux of a metabolic pathway. Xueqin Lv et al. have introduced recent studies on scaffold-free strategies, synthetic artificial scaffolds, and physical compartments for enzyme assembly or pathway sequestration. Moreover, the authors also discuss further the potential applications and challenges of compartmentalized metabolic flux control [9].

In recent years, deep learning has experienced exciting progress in influencing human life, and science was no exception. Our Special Issue covered two interesting topics showing how machine learning can support mass data processing in spectrometry-based metabolomics [10] and promoter designs in *Saccharomyces cerevisiae* [11]. Applications of deep learning in synthetic biology and systems biology are still in the early stage and require standardizations. We all look forward to the upcoming advancements in this field. While computational tools support whole-cell biocatalyst design, parallelization, and miniaturization, which speed up the characterization of mutants. Still, the goal has to be a knowledge-based design and a high information content phenotyping.
